# Knowledge about sucking habits. Elaboration, validation and application of an online questionnaire: an exploratory study

**DOI:** 10.1590/2177-6709.31.4.e2625342.oar

**Published:** 2026-09-28

**Authors:** Karen Regina Francisco SILVA, Fernanda Cordeiro RAMOS, Mariana Neder BOMFIM, Maria Vitória Rangel CAMPOS, Lidiane Alipio PEREIRA, Lucas Alves JURAL, Marcela Baraúna MAGNO

**Affiliations:** 1 Universidade de Salgado de Oliveira, Dental School (Rio de Janeiro/RJ, Brazil).; 2 Universidade Federal do Rio de Janeiro, Dental School, Department of Pediatric Dentistry and Orthodontics (Rio de Janeiro/RJ, Brazil).; 3 Universidade Federal de Uberlândia, Dental School (Rio de Janeiro/RJ, Brazil).; 4 Universidade Federal do Rio de Janeiro, Dental School, Department of Prosthetics and Dental Materials (Rio de Janeiro/RJ, Brazil).

**Keywords:** Sucking behavior, Child, Habits, Malocclusion, Knowledge, Comportamento de sucção, Criança, Hábitos, Má oclusão, Conhecimento

## Abstract

**Objective:**

This exploratory study aimed to assess the population’s knowledge about sucking habits (SHs).

**Material and Methods:**

An observational study was developed using data collection through an electronic questionnaire, which had previously been elaborated and validated and was sent to the general population (over 18 years of age) through social media and message applications. The validity of the convergent and discriminant construct, reliability, and internal consistency of the data were evaluated in order to validate the questionnaire’s acceptability. The influence of sociodemographic factors - being a mother/father and SH history concerning the participants’ average knowledge on the issue - was evaluated through linear regression (α=0.05).

**Results:**

The questionnaire showed good acceptability and discriminating properties (p<0.05), strong construct validity (r=0.62), satisfactory reliability (Cronbach’s alpha =0.72), and replicability (ICC=0.89). In total, 294 participants were included and presented an average of 5.21 [± 2.04] correct answers. After linear regression, participants who only worked in health areas and who had only one child continued to present greater knowledge about SHs (p<0.05).

**Conclusion:**

It can therefore be concluded that the knowledge of the population concerning SH is insufficient and further studies, as well as strategies for education, guidance, and dissemination of information regarding SHs should be conducted.

## RESUMO

**Objetivo:**

O objetivo deste estudo exploratório foi avaliar o conhecimento da população sobre hábitos de sucção (HS).

**Material e Métodos:**

Foi desenvolvido um estudo observacional com coleta de dados por meio de um questionário eletrônico, previamente elaborado e validado, enviado à população geral (maiores de 18 anos) por meio de mídias sociais e aplicativos de mensagens. Para a validação do questionário, foram avaliadas a aceitabilidade, a validade convergente e discriminante do construto, a confiabilidade e a consistência interna dos dados. A influência de fatores sociodemográficos - ser mãe/pai e histórico de HS, com relação ao conhecimento médio sobre o tema - foi avaliada por meio de regressão linear (α=0,05).

**Resultados:**

O questionário apresentou boa aceitabilidade e propriedades discriminantes (p<0,05), forte validade de construto (r=0,62), confiabilidade satisfatória (alfa de Cronbach =0,72) e replicabilidade (ICC=0,89). No total, 294 participantes foram incluídos e apresentaram uma média de 5,21 [± 2,04] respostas corretas. Após regressão linear, apenas aqueles que trabalhavam na área da saúde e que tinham apenas um filho apresentaram maior conhecimento sobre o HS (p<0,05).

**Conclusão:**

Conclui-se que o conhecimento da população sobre o hábito de sucção é insuficiente e que estudos adicionais, bem como estratégias de educação, orientação e disseminação de informações sobre o assunto, devem ser realizados.

## INTRODUCTION

Sucking and swallowing have been present since intrauterine life and their movements can already be observed between the 13^th^ and 16^th^ weeks of intrauterine life. The suction reflex movements provide an adequate growth of the craniofacial structures and dental arch, which in turn develop the functions of the stomatognathic system, such as breathing, swallowing, and speech.^1^

These habits are classified as nutritive when related to natural or artificial breastfeeding, and nonnutritive, such as thumb sucking and pacifier use, when performed without nutritive purposes to provide emotional comfort.^2^ According to the World Health Organization (WHO), exclusive breastfeeding is recommended through the first six months of the baby’s life and as complementary feeding up to 24 months, or more^3^ in some cases, as it confers numerous benefits, such as the prevention of malocclusion, atypical swallowing, and temporomandibular disorders, as well as acts in the protection of harmful habits.^4^ Additionally, breastfeeding is associated with protection against respiratory diseases,^5^ infant mortality,^6^ among other aspects.

Even after having enough food, the baby continues to suck the mother’s breast to satisfy his/her psychological need.^7^ Thus, sucking without nutritional purposes could be considered a normal part of fetal and neonatal development, and the reflex of suction continues until approximately 12 months of life. After this stage of development, the baby has already learned to eat and no longer needs the suction reflex to obtain nutrition.^8^ However, the psychological need could persist for a longer period of time, or appear in specific moments, when the child is unhappy, tired, about to fall asleep, when there is early withdrawal from breastfeeding, or even when he/she passes through a period of regression to an earlier emotional stage of development.^9^

Non-nutritive sucking, such as a pacifier and digital suction, when performed with adequate frequency, intensity, and duration, does not interfere with muscle and orofacial growth,^10^ and can even present analgesic properties.^11^ However, when performed with great frequency, intensity, and duration, in addition to individual factors, such as genetic predisposition,^12^ non-nutritive sucking can alter normal patterns of facial growth and the development of stomatognathic functions.^7^

Malocclusion, atypical swallowing, and mouth breathing, generated by the habit of non-nutritive sucking, can have a negative impact on quality of life, not only from an oral and physiological point of view, but psychosocial as well.^13^ According to International Association of Pediatric Dentistry (IAPD) the management of the developing dentition includes, among other factors, recognition and identification of risk factors, seeking to contribute to proper dentofacial development and stable, acceptable (functional, esthetic) occlusion in the permanent dentition.^14^ Hence, it is important for the population to understand these habits so that they can comprehend the etiology of some oral changes. There are several studies concerning oral deleterious habits and their relationship with oral changes; however, this issue does not reach a large part of the general population. Therefore, the present study aimed to design and validate a questionnaire on sucking habits (QSH) and, through this tool, assess the knowledge of the general population on this subject.

## MATERIAL AND METHODS

This study was submitted to and approved by the Research Ethics Committee with Human (CAAE37293220.6.0000.5289). 

### QUESTIONNAIRE DEVELOPMENT AND VALIDATION

An electronic questionnaire with the objective of identifying participants’ knowledge regarding the risk factors and benefits related to sucking habits (SH) and their influences on the development of oral changes in children was elaborated and distributed through an access link. The validation process was divided into three stages:

*1. Drafting the questionnaire*: 26 multiple choice questions were divided into five blocks: (A) Eight questions referring to the participant’s personal information; (B) Three questions related to non-nutritive sucking habits (NNSH), exclusively for participants who had children; (C) Three questions related to a history of malocclusion due to NNSH, frequency of seeking dental care, and sources of information about NNSH; (D) Ten affirmations related to SHs; and (E) One question concerning ethical aspects and one question asking if the participants would like to receive the results of the study.

2. *Evaluation by dentists, lay people, and education professionals*: The questionnaire was evaluated by ten adult individuals (three pediatric dentists, six participants not related to Dentistry, and one specialist in linguistics) for suggestions regarding content and understanding. 

Participants who were considered eligible for this study included pediatric dentists with a Master’s degree or Ph.D.; along with lay people from both genders, with different educational levels, who did not work in the field of Dentistry, regardless of whether or not they had children. These participants were recruited by convenience sampling from the research team’s contacts, with diverse sociodemographic characteristics and personal experiences, to ensure a heterogeneous evaluation. The participants were asked to make remarks, modifications, and suggestions, and to point out any potential flaws. One linguistics specialist evaluated the questionnaire in terms of its orthography, ambiguity, interpretation, and clarity.

The questionnaires, together with the participants’ comments, were returned to the researchers, and a consensus about the suggestions made by the pediatric dentists, participants who did not work in the field of Dentistry, and the specialist in linguistics was gathered. Considerations were evaluated, and those considered pertinent were incorporated into the questionnaire.

3. *Validation of the questionnaire*: The version of the questionnaire with the incorporated considerations was answered by 40 participants. At this moment, participation eligibility included adults, both men and women, regardless of whether or not they had children, those who did not work in the field of Dentistry, with different educational levels. Dental assistants and technicians, as well as dental prosthesis technicians, were excluded. The questionnaires were applied virtually to the same participants at two different times, with an interval of 15 days between the two applications (test and retest).

### QUESTIONNAIRE APPLICATION

Once the QSH was validated, it was distributed to the target audience of the study via email, WhatsApp, Instagram, and Messenger during the month of February 2021. A convenience snowball sample method was conducted, where the sample was collected through link-tracing sampling, or respondent-driven sampling, to collect data on population members. The seeds for respondent-driven sampling were chosen through convenience sampling in the individual and group contact list of research authors. The participants were asked to identify other possible participants, who in turn were asked to identify other possible participants, and so forth, for the purpose of obtaining a nonprobability sample or to build a framework from which the sample could be formulated.^15^

Those participants who did not answer all questions of the questionnaire and who were under 18 years of age were excluded. 

The researchers considered the block of answers related to knowledge about SHs (block D) to be correct. The answers considered to be incorrect, or “I don’t know”, received a score of 0, while the answers considered to be correct received a score of 1. The final average of correct answers was calculated by adding the scores of each question. Since the QSH of block D consisted of 10 affirmations about SHs, the knowledge score could vary from 0 to 10.

### STATISTICAL ANALYSIS

#### Statistical analysis - Questionnaire validation

Acceptability was assessed using the proportion of individuals who did not complete the questionnaire, while the parametric distribution of data was assessed using the Shapiro-Wilk test.

The convergent construct validity was tested through the correlation between the mean of correct answers from the QSH and the self-knowledge judgment about SHs, using Spearman’s correlation coefficient: correlation coefficients <0.4 = weak magnitude correlation; ≥0.4 to <0.5 = moderate magnitude; and ≥0.5 = strong magnitude.^16^ The validity of the discriminant construct was investigated by comparing the mean of correct answers from the QSH between the groups who worked in and who did not work in the health field, using the t-test for independent samples.

Reliability was assessed according to the criteria of internal consistency and temporal stability (agreement between repeated test-retest measures). The intraclass correlation coefficient (ICC) was calculated for the total score, and Cronbach’s alpha was calculated for the total questionnaire, as well as after excluding individual items, together with the correlation coefficients between the items and the mean score. Evidence of ICC and Cronbach’s alpha reliability was considered to be ≥0.7.^17^

#### Statistical analysis - Questionnaire application

Descriptive statistics were applied to express the results in means and standard deviations. The parametric distribution of the data was tested using the Shapiro-Wilk test, and the inferential analysis included the Mann-Whitney and Kruskal-Wallis test (with comparison between pairs using the Bonferroni test) for intergroup comparisons. The chi-square test was applied to assess the association between having children and a source of information about NNSH. Variables with significance lower than 0.05 and with a minimum number of 10 cases per category were the parameters used to remain as adjustment variables in the multivariate model that tested the influence of the exposure factors on the knowledge about SHs (dependent variable). VIF (variance inflation factor) was evaluated to verify collinearity between the independent variables and the possibility of a model with multiple simultaneous exposures.

In both statistical analyses (questionnaire validation and main study), the level of significance was 5% (α=0.05). The data were tabulated and analyzed in IBM SPSS Statistics for Windows for all analyses (IBM SPSS, version 21.0, 2012; IBM Corp., Armonk, NY).

## RESULTS

### QUESTIONNAIRE VALIDATION

The QSH obtained a response rate of 89.4% and 97.6% in the test and retest, respectively, indicating good acceptability of the instrument. The convergent construct validity between the mean of correct answers from the QSH and the self-judgment of knowledge was r=0.62, while the validity of the discriminant construct showed that there was a significant difference (p<0.05) in the level of knowledge between the groups who worked in and who did not work in the health field.

The internal consistency showed a satisfactory degree for the instrument as a whole. Cronbach’s alpha for the questionnaire was 0.72, whereas the alpha values for one-by-one item excluded ranged from 0.681 to 0.754. The ICC found for the total score was 0.89. The mean scores of the QSH during the first (test) and second (retest) administrations of the questionnaire were 5.05 (standard deviation [SD]=2.4) and 5.5 (SD=2.4), respectively.

Table 1 shows the numerical results for the validation of the QSH, and Table 2 shows the sample distribution of the participants involved in the validation of the questionnaire.

**Table 1:** QSH validation results.

	Test applied	Results	p value
Acceptability			
Test	% of participation	89.4%	
Retest		97.6%	
Construct validity			
Convergent	Spearman Correlation	r 0.62	
Discriminant	Independent test t	MD 2.47	0.004
Confiability			
Intra-participant concordance	ICC	0.89 [0.79, 0.94]	<0.001
Intern concordance	Cronbach ALPHA	α = 0.72	

r: Spearman’s correlation coefficient; MD: Mean difference; α: alpha.

**Table 2:** Sample distribution during test-retest (n=40)

	n	Mean ± SD (correct answer)	p value
Age (Mean ± SD)	35.9 ± 14.8		
Sex			0.715^t^
Men	11 (27.5%)	4.8 ± 2.5	
Women	29 (72.5%)	5.1 ± 2.4	
Others	0		
Schooling			0.973^A^
Until complete high school	15 (37.5%)	5.1 ± 1.7	
Complete higher education	11 (27.5%)	4.9 ± 2.5	
Post graduation	14 (35%)	5.1 ± 3.1	
Health area			0.004^t^
Yes	10 (25%)	6.9 ± 2.8	
No	30 (75%)	4.4 ± 1.9	
Have children			0.62^t^
Yes	24 (60%)	5.2 ± 2.3	
No	16 (40%)	4.8 ± 2.6	
NNSH in your son			0.003^t^
Yes	16 (66.7%)	4.25 ± 1.9	
No	8 (33.3%)	7.1 ± 2.1	
Malocclusion due to NNSH			0.189^A^
Didn’t had NNSH	13 (32.5%)	4.5 ± 2.2	
Yes, due NNSH	9 (22.5%)	5.6 ± 3.1	
No	7 (17.5%)	6.6 ± 1.9	
Dont know	11 (27.5%)	4.4 ± 2.1	
Total correct answer - Test		5.05 ± 2.4	
Total correct answer - Retest		5.5 ± 2.4	

t Independent t test; ^A^ ANOVA; NNSH = Non nutritive sucking habits.

Boxes 1 and 2 present the QSH with affirmations and responses in Portuguese and non-validation English, respectively, and theorical reference adopted^3^ ^,^ ^18^ ^-^ ^23^. 

**Box 1:** Affirmations and responses, in Portuguese, and theorical reference adopted.

Afirmação	Resposta	Referência
P1. A necessidade de sucção de um bebê começa ainda no período intrauterino (na barriga da mãe).	Concordo	Corrêa^18^ (2011)
P2. A necessidade de sucção de um bebê diminui com o passar dos primeiros meses e, aos 6 meses, não há mais essa necessidade.	Discordo	Corrêa^18^ (2011)
P3. Bebês que receberam aleitamento materno (mamaram no peito) por mais tempo têm maior chance de chupar dedo quando crianças.	Discordo	ABOPed^19^ (2024)
P4. Bebês que chupam chupeta têm maior chance de interrupção da amamentação natural (parar de mamar no peito).	Concordo	Buccini et al.^20^ (2017) and WHO/UNICEF^50^
P5. Chupar dedo ou chupeta intensamente (por um longo período e todos os dias) pode causar problemas de mordida na criança.	Concordo	ABOPed^19^
P6. Chupeta ortodôntica tem menor chance de causar problemas de mordida do que a chupeta convencional.	Discordo	ABOPed^19^
P7. Amamentar no seio da mãe e chupar chupeta, durante a vacinação, pode diminuir a dor do bebê durante a injeção.	Concordo	Ministério da Saúde^21^
P8. Crianças que chupam chupeta por muito tempo têm maior risco de serem fumantes quando adultos.	Concordo	Ferreira et al.^22^ (2015)
P9. Chupar chupeta pode estar associado a problemas no sono das crianças, como dificuldades para pegar no sono ou acordar durante a noite.	Concordo	Messayke et al.^23^ (2020)
P10. Para prevenir problemas de mordida nos dentes permanentes (dentes de adulto) em crianças que chupam dedo ou chupeta, é preciso que este hábito seja removido antes dos dentes permanentes nascerem.	Concordo	ABOPed^19^

**Box 2:** Affirmations and responses, in non-validation English, and theorical reference adopted.

Affirmation	Answer	Reference
P1. The need for sucking of the baby begins during in the intrauterine period (in the mother’s womb).	Agree	Corrêa^18^ (2011)
P2. A baby’s need for sucking decreases over the first few months and, by 6 months, there is no longer this need.	Disagree	Corrêa^18^ (2011)
P3. Babies who were breastfed for a longer time are more likely to suck their thumbs as children.	Disagree	ABOPed^19^
P4. Babies who suck a pacifier are more likely to interrupt natural breastfeeding (stop breastfeeding).	Agree	Buccini et al.^20^ (2017) e WHO/UNICEF^50^
P5. Sucking a thumb or pacifier intensely (for a long period of time and every day) can cause biting problems in the child.	Agree	ABOPed^19^
P6. Orthodontic pacifiers are less likely to cause biting problems than conventional pacifiers.	Disagree	ABOPed^19^
P7. Breastfeeding at the mother’s breast and sucking a pacifier during vaccination can reduce the baby’s pain during the injection.	Agree	Ministério da Saúde^21^
P8. Children who suck pacifiers for a long time are at greater risk of becoming smokers as adults.	Agree	Ferreira et al.^22^ (2015)
P9. Sucking a pacifier can be associated with sleep problems in children, such as difficulty falling asleep or waking up during the night.	Agree	Messayke et al.^23^ (2020)
P10. To prevent biting problems with permanent teeth (adult teeth) in children who suck thumbs or pacifiers, this habit must be removed before permanent teeth erupt.	Agree	ABOPed^19^

### QUESTIONNAIRE APPLICATION

The QSH was accessed by 327 individuals, of whom 7 were excluded because they did not answer the questionnaire completely. In addition, 26 respondents were excluded because they were under 18 years of age. Finally, of the 294 participants, most were women (74.5%), from Southeast region of Brazil (67.3%), from 18 to 39 years of age (69%), with an educational level of complete high school (49.3%) (Table 3), and with an average of 5.21 (±2.04) correct answers. The average time spent by the participants answering the 26 questions from the QSH was 5 minutes.

**Table 3:** Sample distribution in the questionnaire application (n=294).

	n	Mean ± SD	p value	Linear regression	
				B	p value
Country region			0.454^KW^	-	
North	57 (19.4%)	5.0 ± 2.2			
Northeast	10 (3.4%)	5.2 ± 1.7			
Midwest	16 (5.4%)	6.2 ± 1.9			
Southeast	198 (67.3%)	5.2 ± 2.0			
South	13 (4.4%)	5.3 ± 1.8			
Age			0.01^MW^	-0.53	0.107
18 to 39 years	203 (69%)	5.4 ± 2.1			
40 years or more	91 (31%)	4.8 ± 1.8			
Gender			0.582^MW^	-	
Men	75 (25.5%)	5.1 ± 2.1			
Women	219 (74.5%)	5.2 ± 2.0			
Other	0	-			
Schooling			0.006^KW^	0.13	0.46
Until complete high school	145 (49.3%)	5.0 ± 1.8			
Complete higher education	59 (20.1%)	4.8 ± 2.1			
Post graduation	90 (30.6%)	5.8 ± 2.2			
Looking for dental care			0.136^KW^	-	
Never	2 (0.7%)	4 ± 4.2			
In case of problems	95 (32.3%)	4.8 ± 2.0			
Every 6 months	73 (24.8%)	5.3 ± 1.9			
Every year	87 (29.6%)	5.6 ± 2.2			
In treatment / demand frequently	37 (12.6%)	4.9 ± 1.6			
Work in health area			<0.001^MW^	-1.45	<0.001
Yes	117 (39.8%)	6.1 ± 1.9			
No	177 (60.2%)	4.6 ± 1.9			
Work or study in dentistry area			<0.001^MW^	-0.28	0.61
Yes	61 (20.7%)	6.2 ± 1.8			
No	233 (79.3%)	5.0 ± 2.0			
Malocclusion due NNSH			0.036^MW^	0.06	0.68
Didn’t had NNSH	82 (27.9%)	5.3 ± 2.1			
Yes, due NNSH	57 (19.4%)	5.7 ± 2.0			
No	103 (35%)	5.2 ± 2.0			
Don’t know	52 (17.7%)	4.6 ± 1.9			
Have son			0.975^MW^	-	
Yes	147 (50%)	5.3 ± 1.9			
No	147 (50%)	5.2 ± 2.1			
Son with NNSH (n=147)			0.461^MW^	-	
Yes	103 (70.1%)	5.1 ± 1.9			
No	44 (29.9%)	5.5 ± 2.1			
How many children (n=147)			0.003^MW^	-0.66	0.046
1 son	62 (42.2%)	5.8 ± 2.0			
2 sons or more	85 (57.8%)	4.8 ± 1.8			
Conduct about NNSH in your son (n=85)			0.1^KW^	-	
Same conduct in both sons	60 (70.6%)	4.8 ± 1.8			
Avoid in 1st son and allowed in 2nd son	10 (11.8%)	4.2 ± 1.2			
Allowed in 1st son and avoid in 2nd son	15 (17.6%)	5.5 ± 1.8			
Reasons your child underwent NNSH					
Don’t know	11 (3.4%)				
To stop crying	44 (13.8%)				
To sleep	79 (24.7%)				
Fearful situations	5 (1.6%)				
To calm down	55 (17.2%)				
Amusement / Cute habit	0				
Missing parents	1 (0.3%)				
To supplement breastfeeding	15 (4.7%)				
Others	2 (0.6%)				

MW = Mann-Whitney; KW = Kruskal-Wallis.

Figure 1 shows the response rate ‘agree’, ‘disagree’, and ‘I don’t know’ for each affirmation in QSH, block D.

**Figure 1:**
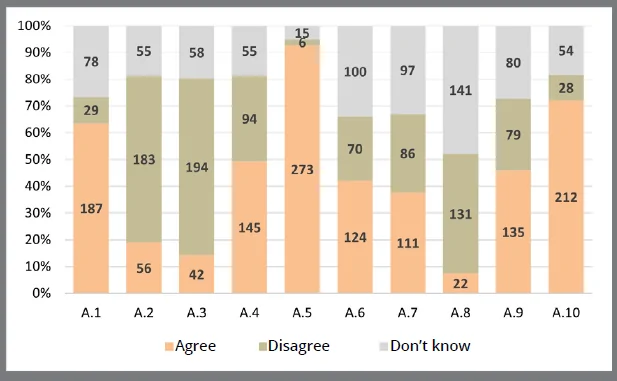
QSH distribution of responses (n=294).

The questionnaire was applied in Portuguese and the non-validated English translation is presented below: **A.1** - A baby’s need to suck begins even in the intrauterine period (in the mother’s womb); **A.2** - A baby’s need to suck decreases over the first few months and, by 6 months, this need no longer exists; **A.3** - Babies who were breastfed for a longer period are more likely to suck their thumb as children; **A.4** - Babies who use pacifiers are more likely to interrupt breastfeeding; **A.5** - Sucking a thumb or pacifier intensely (for a long period and every day) can cause biting problems in the child; **A.6** - Orthodontic pacifiers are less likely to cause biting problems than conventional pacifiers; **A.7** - Breastfeeding and using a pacifier during vaccination can reduce the baby’s pain during the injection; **A.8**- Children who use pacifiers for a long time (many years) have a higher risk of becoming smokers as adults; **A.9** - Sucking on a pacifier may be associated with sleep problems in children, such as difficulty falling asleep or waking up during the night; **A.10** - To prevent biting problems in permanent teeth (adult teeth) in children who suck their thumb or pacifier, this habit needs to be removed shortly before the permanent teeth erupt.

Table 3 describes the absolute frequencies, mean, and SD for sociodemographic characteristics, NNSH during maternity, history of malocclusion due to NNSH, and frequency of seeking dental care. The pesent study showed that the participants between 18 and 39 years of age (p=0.01), who had a postgraduate degree (p=0.006), who were working in areas of health and Dentistry (p<0.001), and who had only one child (p=0.003) tend to have greater knowledge about SHs. Peer comparisons indicate that those with post-graduate degrees show a higher mean of knowledge about SHs, when compared to participants with educational levels of up to complete high school (p=0.01) and complete higher education (p=0.032). They also indicated that those who had malocclusion due to NNSH tend to present a statistically higher mean of knowledge about SHs than do those who “don’t know” about their history (p=0.026).

The corrected linear regression model showed F=5.81, P<0.001 and r^2^=0.165. VIF ranged from 1.09 to 1.48, indicating that there is no multicollinearity in the model. Only working in health areas and having only one child continued to show greater knowledge about SHs.

Mothers and fathers with children who had NNSH reported that the main reasons for performing NNSH are to sleep (24.7%), to calm down (17.2%), and to stop crying (13.8%). Among mothers and fathers with more than one child, 70.6% report that they maintained the same behavior.

In general, 34% of the participants had never received prior information on NNSH, and, among those who had received prior information, the major sources of information reported were: a dentist or health professional (27.9%), TV or internet (22.1%), and in college or at work (21.3%). The chi-square test showed that there is a positive association between having children (being a parent) and receiving information about NNSH through health professionals (p=0.037), and a negative association between having children and receiving information about NNSH in college or at work (p<0.001) (Table 4).

**Table 4:** Association between have a son and source of information about NNSH.

Previous information about NNSH	Total	Son(s)		p value^X2^
		Yes (n=147)	No (n=147)	
Never	102 (34.7%)	44	58	0.09
TV or Internet	65 (22.1%)	34	31	0.67
School, college or work	68 (21.3%)	21	47	<0.001
Friends	28 (9.5%)	13	15	0.69
School of the son	1 (0.3%)	1	0	0.32
Professional of health area	82 (27.9%)	49	33	0.037
Other sources	34 (11.6%)	21	13	0.14

X^2^ = qui-squared test.

## DISCUSSION

Using validated tools is necessary to avoid any error or inability of the researcher when collecting the data and to avoid confusing and incomplete information.^24^ Based on this importance, the QSH validation was carried out in three stages: preparation, evaluation by dentists, lay people and linguistics professionals, and application in two moments (test-retest). The questionnaire showed good acceptability and discriminating properties, strong construct validity, as well as satisfactory reliability and replicability, indicating that the QSH is easy to understand, with clearly defined questions and answer alternatives, along with valid and reliable properties for application among Brazilian adults.

The time required for the 294 participants to answer the 26 questions from the QSH was, on average, five minutes. The short time required can justify the acceptability and excellent adherence of approximately 98% of the participants who opened the link to access the questionnaire. However, it should be remembered that the total number of people who received the access link and did not open it cannot be measured, as the “snowball” method for disseminating the research was used.

After linear regression, age and educational level showed no influence on one’s knowledge of SHs. This finding differs from other studies that have reported associations between age and educational level and knowledge of oral habits,^25^ that younger mothers are more likely to have a poor level of knowledge about their baby’s oral health care,^26^ that women with lower educational levels allow their children to use a pacifier for longer periods (frequency and duration of the habit),^27^ and that mothers with higher educational levels tend to encourage their children to stop these habits.^28^ These discrepancies in the results may be associated with the fact that the present study performed a multivariate linear regression analysis, which allows one to understand the relationship between an outcome variable and a predictor, controlling for the effect of other predictor variables. 

The prevalence of traits of malocclusion ranges from 74.7% to 4.91%, varying in relation to its prevalence and types according to the dentition and aspects related to nationality.^29^ In Brazil, where the present study was carried out, the prevalence of malocclusion in permanent dentitions has been reported to be around 71%.^30^ ^,^ ^31^ Additionally, this condition is even more prevalent in patients with practical SHs.^32^ These reports disagree with the results of the present study, where only 19.4% of the participants reported having malocclusion related to NNSH. This discordance may be related to the fact that malocclusion related to NNSH was assessed through the participants’ self-reports, and self-reporting may be a low-sensitive method for laypeople to diagnosis malocclusion. This aspect could be considered a limitation.

Other studies that evaluated malocclusion through self-reported questionnaires reported a prevalence of this condition between 47%^33^ and 56%.^34^ These reported prevalences are lower than those obtained through clinical examination, but they are still far superior to the prevalence found in the present study. This finding may well be related to undernotification of health conditions when evaluating through self-reporting, and this underreporting can be further aggravated by being a result of others’ assessment (in the present study, the assessment of the caregiver in relation to their child). Studies including QSH and malocclusion clinical examination in Primary Health Care (PHC) units are encouraged.

According to findings from Góes et al.^35^, a wide range of health professionals have shown great interest in NNSH, as these habits can cause changes in the stomatognathic system, which is directly related to the child’s behavior, quality of life,^36^ happiness,^37^ and social relationships.^13^

Many health and dental professionals are interested in this issue, as their knowledge base is commonly focused on this area of study. The authors of the present study suggest that health professionals showed a higher number of correct answers; however, prior studies have both agreed and disagreed with this hypothesis.

The data from the present study indicate that parents who have only one child have more knowledge about SHs. The authors presume that mothers and fathers who have only one child may have more time to search for information on aspects related to breastfeeding and SHs. By contrast, another study reports that mothers who had only one child were more likely to perform early weaning, which could lead to NNSH.^38^ This disagreement may suggest that other sociodemographic aspects not evaluated in the present study (such as working outside the home, family structure, support network, means of accessing information, among others) may work together to determine this knowledge in families with different numbers of children.

As a limitation of the present study, we can mention the convenience sample and the snowball sampling method used to invite participants through social networks. This method became very popular after the COVID-19 pandemic, when sanitary measures hindered traditional research approaches^39^ and online research. Although the number of studies using this methodology increased during the pandemic in several health areas and in several locations worldwide,^40^ ^-^ ^42^ which can be considered a modern, useful, and economical approach to research,^43^ there is still limited information on the use of digital tools and social media in accessing hard-to-reach populations and on the comparability of populations recruited through these and traditional methods.^44^ Thus, new studies using the QSH validated in the present study should be conducted in an in-person format.

Additionally, it is important to highlight that, although the sample included representatives from all of the important occupational groups, it was not necessarily representative of any specific population. Though the present study was not limited to any specific Brazilian region, educational level and the gender of the participants included in the present study’s sample could not be considered a national representative sample. Moreover, to avoid skepticism from the Brazilian population, the present study was defined as exploratory.

It is important to highlight the high rate of “I don’t know” answers to almost all affirmations related to knowledge about SHs, indicating the participants’ shallow knowledge on the issue. Almugairin et al.^28^ reported that mothers present moderate overall awareness; however, a significant gap existed between knowledge and practice. A previous study showed that although parents or caregivers admitted being aware that these habits could be detrimental to teeth, most of them already offered a pacifier to children.^45^ Hence, strategies to prevent SHs should be disseminated. 

The pacifier is a low-price device that is accessible to the majority of the population and has a symbolic association with newborns, representing one of the key items in the baby’s outfit.^46^ According to the Brazilian Society of Pediatrics, a new approach is needed for this population, especially as regards the pros and cons of using a pacifier, including information campaign policies and educational actions.^47^ Brazil has the Brazilian Standard for the Marketing of Food for Infants and Early Childhoods, Nipples, Pacifiers, and Baby Bottles (NBCAL - Law No. 11,265), which is a set of rules that regulate the commercial promotion and labeling of foods and products intended for newborns and children up to three years of age, which has become an important instrument for the control of indiscriminate advertising of foods and childcare products that compete with breastfeeding. 

However, one must bear in mind that health education is a complex issue in its feasibility, requiring a combination of educational and environmental support to achieve its objective.^48^ NBCAL’s monitoring actions must be continuous, with action plans involving government partners and other social actors, in order to achieve an effective evaluation of the policy of promoting healthy eating habits in all stages of life, with breastfeeding at the beginning of this process. In addition, it is necessary to understand the theme, concepts, and aspects that it covers, as well as the association of this practice with communication, information, education, and qualified listening for health promotion to occur.^20^ ^,^ ^49^ The authors believe that collective and individual strategies, as well as the best preparation of health professionals, should be planned to ensure a quality democratization of information about SHs.

## CONCLUSION

According to the result of this exploratory study, it is possible to conclude that the population’s knowledge about SHs can be considered insufficient. Age, educational level, working as a health professional, having a history of malocclusion due to NNSH, and the number of children could influence this knowledge. Quality information needs to reach the general population with information campaigns and guidance from health professionals.

## Data Availability

All data generated or analyzed during this study are included in this published article
